# “*It’s hard to say anything definitive about what severity really is*”: lay conceptualisations of severity in a healthcare context

**DOI:** 10.1186/s12913-024-10892-6

**Published:** 2024-04-19

**Authors:** Mille Sofie Stenmarck, David GT Whitehurst, Hilde Lurås, Jorun Rugkåsa

**Affiliations:** 1 The Health Services Research Unit– HØKH, Akershus University Hospital HF, Lørenskog, Norway; 2 Faculty of Medicine, University of Oslo, Oslo, Norway; 3 Faculty of Health Sciences, Simon Fraser University, Vancouver, Canada; 4 Faculty of Health Sciences, Oslo Metropolitan University, Oslo, Norway; 5 Faculty of Health and Social Sciences, University of South-Eastern Norway, Porsgrunn, Norway

**Keywords:** Priority setting, Health policy, Severity, Thematic analysis, Views of the public

## Abstract

**Background:**

Demand for healthcare outweighs available resources, making priority setting a critical issue. ‘Severity’ is a priority-setting criterion in many healthcare systems, including in Norway, Sweden, the Netherlands, and the United Kingdom. However, there is a lack of consensus on what severity means in a healthcare context, both in the academic literature and in policy. Further, while public preference elicitation studies demonstrate support for severity as a relevant concern in priority setting, there is a paucity of research on what severity is taken to mean for the public. The purpose of this study is to explore how severity is conceptualised by members of the general public.

**Methods:**

Semi-structured group interviews were conducted from February to July 2021 with members of the Norwegian adult public (n = 59). These were transcribed verbatim and subjected to thematic analysis, incorporating inductive and deductive elements.

**Results:**

Through the analysis we arrived at three interrelated main themes. *Severity as subjective experience* included perceptions of severity as inherently subjective and personal. Emphasis was on the individual’s unique insight into their illness, and there was a concern that the assessment of severity should be fair for the individual. The second theme, *Severity as objective fact*, included perceptions of severity as something determined by objective criteria, so that a severe condition is equally severe for any person. Here, there was a concern for determining severity fairly within and across patient groups. The third theme, *Severity as situation dependent*, included perceptions of severity centered on second-order effects of illness. These included effects on the individual, such as their ability to work and enjoy their hobbies, effects on those surrounding the patient, such as next of kin, and effects at a societal level, such as production loss. We also identified a concern for determining severity fairly at a societal level.

**Conclusions:**

Our findings suggest that severity is a polyvalent notion with different meanings attached to it. There seems to be a dissonance between lay conceptualisations of severity and policy operationalisations of the term, which may lead to miscommunications between members of the public and policymakers.

**Supplementary Information:**

The online version contains supplementary material available at 10.1186/s12913-024-10892-6.

## Background

The demand for healthcare services outweighs available resources, and governments face complex dilemmas of healthcare prioritisation [1, 2]. Priority setting in healthcare is an issue in both low- and high-income countries, and in publicly-funded and private healthcare systems [3]. Healthcare systems rely on priority-setting frameworks to guide decision-making, and there is a broad field of research on the principles underpinning these frameworks. In most universal healthcare systems, priority-setting principles are typically centered on cost-effectiveness criteria [1]. ‘Severity’ is another criterion and has been adopted in several countries, including Norway [4], Sweden [5], the Netherlands [6], and the United Kingdom (UK) [7]. Despite the widespread use of severity as a criterion there is a lack of consensus on how to operationalise it.

A severity criterion modifies decision rules in cost-effectiveness analyses, potentially allowing for the recommendation of treatments (for conditions considered to be ‘severe’) that would otherwise not have met cost-effectiveness thresholds. As such, severity has been described as an ethical decision-modifier [8]. In Norway, priority-setting decisions are to be based on the three criteria of health benefit, resources, and severity [9]. The three criteria are intended to be applied throughout the healthcare system, from health policy to the clinical level, and weighed against each other. The severity criterion is operationalised as absolute quality-adjusted life year (QALY) shortfall [4]. The QALY is a health metric that combines quality and quantity of life in a single outcome [10], and ‘absolute QALY shortfall’ represents the expected loss of QALYs due to illness [11]. Other jurisdictions use different operationalisations: the Netherlands operationalise severity as proportional QALY shortfall, calculated by the expected loss of QALYs relative to remaining life expectancy [6]; in the UK, a combination of absolute and proportional QALY shortfall is implemented [7]; and Sweden employs a severity framework which measures severity according to a qualitative ranking of severity levels, from ‘low’ to ‘very high’ [12, 13]. While severity is a common priority-setting criterion, it is evident that there is no consensus on how to operationalise it in policy.

While attempts at defining severity in the academic literature are usually based on QALYs [14, 15], different conceptualisations exist [16–18]. Olsen argues that (at least) four different approaches can be identified: severity understood according to (i) how poor one’s health is; (ii) short remaining lifetime; (iii) poor prognosis; or (iv) the size of the health loss [11]. This academic ambiguity is present also among stakeholders within the healthcare system. Magnussen and colleagues distributed a survey among healthcare personnel, leaders at different levels of the healthcare system, and patient organisations, and find that there is no agreement on what severity means [4]. This lack of a shared understanding further complicates the use of the term in a policy context [11].

Decisions on healthcare prioritisation inevitably involves allocating resources to some groups over others, making priority setting in healthcare contentious [1]. Because the outcome of these decisions is consequential to the public, knowledge of public views is critical [19]. In a literature review of public preference elicitation studies on the relevance of severity in healthcare, Shah demonstrates that severity is considered an important and relevant concern for priority setting across multiple populations [18]. Studies conducted across Norway, Denmark, Finland, the UK, and the US establish support among the general public for severity as a relevant concern [20–24]. Both an Australian, an Icelandic, and a UK study find that general public respondents prefer at least equal priority to the severely ill [25–27]. And in a Canadian survey with general public participants, severity was ranked as the most important concern across all respondent groups [28].

While previous literature has sought to elicit the degree to which the public consider severity to be *relevant*, via methods comprising small-sample focus groups through to population surveys [18], they do not explore what severity is taken to mean. Furthermore, there is a lack of consensus on how to *define* severity (the aforementioned studies apply different definitions, if they provide one at all), whether for the reader or the participants in the respective studies. Broqvist and colleagues adopt a more explorative approach to understanding severity by comparing views of the public on severity levels within the Swedish priority-setting system [12]. They find that the citizenry considers a multitude of different aspects relevant to determining severity, such as physical or psychological impairment, risk of death, and duration of illness. Their findings suggest that severity is interpreted as something more than QALY shortfall—but they do not provide an in-depth exploration of how people *conceptualise* the notion of severity itself.

It is evident that there is ambiguity surrounding severity in health policy, in the priority-setting literature, and in multiple public preference elicitation studies [16, 29]. There is also a paucity of research on how severity is conceptualised by members of the public. To address this latter knowledge gap, we conducted group interviews with members of the Norwegian population and subjected the data from these to a thematic analysis.

## Methods

### Design

This study is part of the SEVerity and PRIority setting in healthcare (SEVPRI) project, which seeks to explore the general public’s views on severity. Other phases of the SEVPRI project comprise a Q-methodology study to examine accounts of severity and locate shared viewpoints [30], and a cross-sectional survey study to explore the prevalence of different views in a representative sample of the Norwegian population [31]. In this article, we present an analysis of group interview data conducted with members of the public as part of the SEVPRI project. A qualitative design was considered best suited to gain a nuanced and in-depth understanding of how lay people conceptualise severity. Data was collected through group interviews across Norway, in the format of open conversations, that were transcribed and analysed thematically [32].

We anticipated that approximately 60 participants were needed to reach saturation, understood as data redundancy [33] in that new data becomes repetitive of what has been expressed in previous conversations [34]. To ensure a diversity of perspectives, we sampled purposively to achieve representation of different demographics, including age, education level, socioeconomic background, health status, and region. Ahead of data collection, a conversation guide was designed with an outline of the format of the conversations, including an introductory text to introduce participants to the topic of severity, and a topic guide on potentially relevant topics for discussion (see Supplementary Material 1). The introductory text explained, in lay terms, the three priority-setting criteria used in the Norwegian healthcare system, and that particularly severe conditions can be prioritised. The text also stated that our purpose was to explore participants’ subjective viewpoints, and that all thoughts, perceptions, and input was welcome. The presentation was kept brief to avoid influencing participants’ views or priming them on certain perspectives. It also emphasised that there are no right or wrong answers on this topic, and that health personnel, health economists, and philosophers discuss what severity means.

We aimed to identify the breadth of perceptions on severity, and conversations were therefore not moderated to reach consensus. Rather, the facilitators sought to capture the various perspectives participants held by starting the conversations with an open question, and allowing participants to discuss freely and as uninterrupted as possible. This open conversation style (see Supplementary Material 1 for details) was supported by the topic guide to ensure the same topics were covered across the conversations. The topic guide contained possible attributes of severity highlighted in the literature [16]. This included issues deemed to impact the severity of conditions, such as someone’s age, risk of death, and pain, which we could use to prompt participants if they, for example, considered that risk of death or someone’s age made a condition more or less severe. The conversation format, introductory text, and topic guide were piloted with a user panel at Akershus University Hospital, consisting of eight people from different demographic backgrounds. Following positive feedback and minor linguistic edits, data collection commenced.

### Data collection

Participants were recruited via SEVPRI’s social media accounts (Facebook and Twitter), sharing a link to an online recruitment platform. Recruitment posters were hung in shops and on lampposts in Oslo, as well as in the waiting rooms of general practitioners in Oslo and Bergen. The recruitment period was February to July 2021. We began sampling widely across the population. Due to SARS-CoV-2 pandemic restrictions, we first sampled for online conversations, from February to March 2021. Once restrictions were lifted, we began to sample for in-person conversations, sampling for these from April to July 2021. To achieve the desired representation of participant characteristics, sampling became increasingly more targeted (e.g., seeking out male participants when the sample became over-represented by women). In compliance with SARS-CoV-2 pandemic restrictions, the first 14 conversations (February to March 2021) were conducted online, using Zoom [35]. The final seven conversations were conducted in-person following the lifting of restrictions (May to July 2021). In-person conversations were conducted across five locations (Oslo, Bergen, Trondheim, Tromsø, and Alta). Details of conversation format and participants are provided in Table 1. Group size was determined based on two considerations: (i) enough participants to have a meaningful discussion, and (ii) not too many participants, to allow everyone to voice their views, with time to explore the depths of these views. The nature of online meetings—with time lags, less non-verbal communication, and other digital challenges—made these challenging to moderate with larger groups. Online conversations were therefore conducted with a minimum of two participants and a maximum of four. The in-person conversations had an upper limit of eight participants. Participants received a universal gift card as compensation for participation, with NOK 250 (∼€23) for participation in online conversations and NOK 500 (∼€45) for in-person conversations, as these required travel to the meeting locale.

**Table 1:** Overview of conversation format and number of participants (not including facilitator)

Conversation	Format	Number of participants
C1	Online	2
C2	Online	2
C3	Online	2
C4	Online	2
C5	Online	2
C6	Online	2
C7	Online	1^a^
C8	Online	3
C9	Online	2
C10	Online	2
C11	Online	2
C12	Online	3
C13	Online	3
C14	Online	2
C15	In-person	2
C16	In-person	5
C17	In-person	4
C18	In-person	4
C19	In-person	5
C20	In-person	3
C21	In-person	6

^a^ In conversation #7 (C7), a second scheduled participant did not attend. SEVPRI's Principal Investigator participated in the conversation with the participant; the Principal Investigator’s comments were not included in the analysis

We considered that data saturation had been reached after 21 conversations, with a total of 59 participants. Participant demographics are presented in Table 2. The conversations lasted approximately two hours when online and three hours when in-person. The lead author (MSS) facilitated ten of the conversations and was present in another five. As data collection for SEVPRI was a joint effort by the research team, the remaining six conversations were conducted by two other, non-author members of the SEVPRI research team (Mathias Barra and Odd Borgar Jølstad). All conversations were audio-recorded and transcribed verbatim by members of the research team (mainly by the lead author, MSS), in Norwegian.

**Table 2:** Participant demographics from questionnaires. Values are numbers (percentages)

Characteristic	Participants (n = 59)
Age category (years)^a^	
18–30	9 (15)
31–50	13 (22)
51–66	24 (41)
67+	11 (19)
No response	2 (3)
Gender	
Female	38 (64)
Male	19 (32)
Other/prefer not to say	2 (3)
Do you consider yourself religious or spiritual?	
Religious and/or spiritual: active in a congregation	11 (19)
Religious and/or spiritual: not active in a congregation	14 (24)
Neither religious nor spiritual	33 (56)
No response	1 (2)
What is your highest completed education level?	
Elementary/Upper secondary (up to 19 years of age)	9 (15)
Undergraduate degree/Apprenticeship	21 (36)
Graduate degree/PhD	27 (46)
No response	2 (2)
Have you or anyone you know well had severe illness?^b^	
Transient	18 (31)
Chronic	30 (51)
Deadly outcome	42 (71)
No response	0 (0)
How do you view your own health?	
Very good/Good	37 (63)
Just fine	15 (25)
Bad/Very bad	6 (10)
No response	1 (2)

^a^ Age was given in one of the listed age brackets

^b^ Categories are not mutually exclusive

All participants were emailed an information and consent form ahead of the conversations (see Supplementary Material 3), and asked to read this. Before in-person conversations, participants used these forms to give written consent, and confirmed consent orally. In online conversations, oral consent was given. Participants were informed that they could withdraw at any stage, but none availed of this. Participants were also informed that their contributions would be kept anonymous, and that transcriptions of the conversations would be stored securely according to Data Protection Officer regulations at Akershus University Hospital.

Once the introductory text had been read to the participants, conversations were initiated with an open question, asking: *“what does severity mean to you? Feel free to start with the first associations you have”* [English translation]. Each participant was given the opportunity to respond, followed by a group conversation to explore the various views that emerged as well as items from the topic guide. The facilitators encouraged participants to speak freely, and let views participants spontaneously brought up lead the conversations. As stated above, the online sessions had fewer participants than those conducted in-person, but remained focused on encouraging participants to speak freely and openly in open conversation with each other. The format of open conversation allowed participants to discuss and reflect on each others’ views, so that—whether participating in smaller or larger groups—they could share their intuitive thoughts and, as they reflected on these and discussed them with other participants, more considered judgements on the various topics could emerge.

Participants were probed to expand or clarify on what they said. For example, a facilitator could say: *“you said that age seems relevant to you, what do you mean by that?”*. At times, facilitators would also explore what was expressed by providing examples of how particular views would translate into real-world situations. For example, if a participant suggested age was relevant, the facilitator could ask: *“is the condition more severe if it affects a young rather than an old person?”*, or if they suggested desert is relevant to severity, they could ask: “*would you say lung cancer is more severe in a non-smoker, than in someone who has smoked their whole life?”*. The same examples were used throughout the conversations, to ensure a standardised approach.

Following the conversation, participants in the in-person groups completed a questionnaire that asked questions about socioeconomic status, health status, and situations that may have affected their views on severity (see Supplementary Material 2). Online participants completed the same questionnaire over the phone with the facilitator.

### Ethics

The Regional Committee for Medical and Health Research Ethics deemed the SEVPRI study outside the remit of the Norwegian Health Research Act (ref. no. 186,284). Ethical approval was granted by the Data Privacy Officer at Akershus University Hospital Trust following a detailed Data Protection Impact Assessment (*PVO. Nos 20_200* and *21_200*). Throughout the study, we have adhered to all relevant ethical guidelines, specifically the Guidelines for Research Ethics in the Social Sciences and Humanities [36] and the Guidelines for Research Ethics and Scientific Assessment of Qualitative Research in Medicine and Healthcare [37]. All names of people and organisations have been deleted or altered.

### Analysis

The data were subjected to qualitative thematic analysis of repeated cycles of induction and deduction [38]. Data were stored and coded using NVivo (release 1.6.1). The analysis was conducted in four stages combining different analytic techniques, as outlined in Table 3. Our analytical approach is based on systematic text condensation, inspired by Giorgi’s phenomenological analysis [32, 39]. This approach was considered well-suited for our purposes: we wished to explore the concept of severity in-depth, and to both garner detailed understandings of our participants’ views on severity and to identify certain common themes across their individual views. Each stage was led by the lead author (MSS), with contributions from and discussions with all co-authors. In step 2, three authors (MSS, JR, HL) separately read three transcripts and identified potential codes, which were then compared and developed into a coding framework. When conducting qualitative analyses it is important to identify and seek to overcome potential preconceptions the researchers might hold. In this regard, it was helpful that the authors come from different academic backgrounds (spanning economics, medicine, and sociology). Furthermore, while all authors were involved in the analytical process through several rounds of reflexive interpretation, only the first author (MSS) took part in data collection. As such, the material was new to three of four authors, who could question and challenge the development of codes and themes throughout the analytical process. The aim of the analysis was to elicit the breadth of views expressed in the conversations to identify broad themes across participants, rather than individual views. As such, the themes presented below do not represent specific groups of participants and one participant’s contribution might fall into more than one theme, and participants might be aligned with more than one theme. We use quotes from the conversations to illustrate how the themes were developed, and these are identified by an alias name and a code (C1 through C21) indicating in which group conversation the quote was collected from. Furthermore, to ensure we captured the breadth in the data, we paid attention to negative cases [40], i.e., viewpoints which were only expressed by one participant and not identified elsewhere in the data.

**Table 3:** The four stages of analysis

Stages	Description of the analytical process
1: From chaos to codes: Read-through	Getting familiar with the data by reading through all transcripts. Note-taking using mind-maps to record topics for potential codes.
2: Coding the material: Deductive-inductive cycles	Three authors (MSS, JR, HL) independently coding three manuscripts to ensure quality and congruence of coding. Subsequently coding all transcripts, adapting the codebook as necessary. Dynamically developing codebook during the coding process (inductive).
3: From code to meaning: Identifying themes	Studying the codes in isolation and in conjunction with each other, searching for themes. Creating mind maps of potential themes and identifying if and how codes fit within these.
4: From de-contextualisation to recontextualisation: Descriptions	Connecting the themes to broader body of literature, looking for connections within and between themes. Recontextualising by returning to transcripts to consider if themes reflect what participants discussed. Writing out narrative within themes.

In Stage 3 we arrived at three themes, which we see as representing three interrelated conceptualisations of severity: *Severity as subjective experience*; *Severity as objective fact*; and *Severity as situation dependent*.

## Results

As a backdrop to the results, it was evident that the participants had a lot to say about severity and appeared eager to share their views, and the conversations yielded nuanced and differing perspectives. Following the opening question on what severity meant to them, participants spontaneously associated the term with a multitude of issues. These issues ranged from the age of the patient, their risk of death, and the acuteness of their condition, to the potential stigma associated with a condition and how illness affects their next of kin. When discussing severity, they also touched on issues such as hope, fear, desert, and pain. Participants seemed reluctant to describe any condition or situation as ‘non-severe’. When prompted to specify such circumstances, a few participants volunteered examples such as passing knee injury or cosmetic surgery. In general, there seemed to be a reluctancy throughout the conversations to specify any conditions as definitively without potential of being severe. As discussions evolved, one participant could express views that are captured by more than one of the themes we present next.

### Severity as subjective experience

A common topic in the conversations focused on how severity related to the individual’s experience of their situation and illness. As such, severity was expressed as an inherently subjective and personal notion, and no condition could be considered severe (or not) until experienced by the individual as such. Severity was portrayed as something intrapersonal which should be decided by the individual.

Jacob (C21): ‘I think of severity as a very subjective description of how you experience a condition.’

Lisa (C19): ‘Severity is an individual question and it’s an individual assessment.’

It appeared that severity could not be implied from a diagnosis or characteristics of a condition, but from how the patient experiences it. The severity of a condition, such as asthma or a broken leg, might vary between individuals suffering from it, depending on how they perceive their situation. When responding to the opening question, Marianne (C18) pointed to this notion of severity as relative:

All illness is very subjective. What feels severe? For some it’ll be catastrophic to break a leg and immediately feels very severe, if that person thinks that right now my life is ruined because I broke my leg. While for some it’s severe [only] when you’re on your deathbed.

Simon (C2) further argued that severity related to how the individual perceives their situation. Therefore, neither policymakers nor healthcare professionals could understand the severity of a condition the way a patient does:

To me that [illness that effects quality of life] would be a severe disease. Even if it wouldn’t have been defined as a severe disease from the authorities or from the healthcare system it would…for me it would be a severe disease because it keeps me from doing, or being part of, of things, so then it’s severe for me.

Anna (C15) suggested the same by referring to schizophrenia and argued that outsiders cannot fully understand the implications of such a disorder. The power of defining its severity should therefore not rest with doctors, academics, or policymakers, but with the patient:

You probably have little understanding of the severity [of schizophrenia] if you haven’t felt it in your own body.

Some expressed skepticism towards a standardised, “one-size-fits-all” approach. Susanna (C19) suggested that, following the different interpretations of severity that had been discussed in the conversation, guidelines and standards could not account for the complexity of severity:

I don’t see how one could set standardised routines to evaluate severity […] With everything we’ve touched on today, so many factors playing into what severity is, I don’t see how one could make a framework that would fit the best for the majority [of situations]. I’m sure there are some sharper minds than mine who can imagine one, but illness and health and severity is as…I mean, there are as many expressions of that as there are people and conditions combined.

Given the emphasis on the individual’s right to decide what is severe for them, external determination of severity was perceived to impose a form of injustice on the individual. Fair decisions about severity should therefore be done in a manner that is fair to the individual:

Jenny (C18): ‘For me, I think I want ownership of my severity. [Severe illness] isn’t something where someone else can say it’s not dangerous, it’ll pass. I think there’s too much of that. It’s about respecting the other’s severity […] It’s about taking the other’s severity seriously. We can’t define it away.’

Melissa (C20): ‘Depriving people of the subjective experience of severity…you can’t take that away from people. [The subjective severity] is always there. And that’s what the healthcare system has to deal with. The severity that the individual experiences in their situation.’

### Severity as objective fact

In other parts of the conversations, severity was conceptualised from an extrapersonal position, independently of individual experience. The severity of, for example, a stroke appeared to depend on elements of that condition, such as risk of death or prognosis. If deemed severe, a stroke would be equally severe for anyone suffering one. Eric (C13) explained that he has a tendency to overestimate the severity of his ailments, and suggested his judgement might not correspond with the “real” severity of his situation:

You could say that I probably have a bit different pain tolerance than my wife. To be completely honest I’m a bit more of a wimp. And I’ve probably spent more time at the doctor’s than I strictly speaking needed to. And that’s a bit of a shame too because then I take up time that maybe they could have spent on people who were really ill […] I probably experience it as more severe and painful than what it really is.

Eric seemed to suggest that while he might feel that something is severe, each condition has an objective level of severity, independent of his own assessment.

*Severity as objective fact* centered on the idea of set criteria and that the severity of a condition depends on whether such criteria are fulfilled. Participants did not agree what such criteria should be. Some volunteered examples such as prognosis and chronicity, and suggested conditions with good prognoses were less severe than those with poor prognoses, or that chronic conditions were more severe than non-chronic ones. There were also suggestions that severity could be considered along some form of scale, where the severity of a condition might be seen to increase the lower one’s age, or the more pain one has, or by the degree of loss of function.

Sandra (C19): ‘The younger, the more severe a condition should be considered to be.’

Thomas (C2): ‘Severity is first and foremost the degree of ailment and the duration and the loss of functioning.’

The emphasis within this conceptualisation of severity was not which criteria might be employed to determine severity, but rather the notion that severity must be determined in an objective manner, based on measures beyond a patient’s subjective experience and evaluation.

The notion of severity as extrapersonal appeared central to this criteria-based conceptualisation, with emphasis on health outcomes within and across patient groups. This notion often arose in response to subjective conceptualisations of severity. Peter (C14), for instance, argued that individual experience insufficiently describes severity, and suggested that applying subjective interpretations in a healthcare setting would be inappropriate:

If we’re talking about a definition of severity then those subjective things can’t be included. Even if I think that it might be experienced as severe for some…but if you’re going to define it, I don’t think that should be included.

Building on this, several participants suggested that a subjective assessment of severity would also be impractical in a broader healthcare context:

Jon (C18): ‘It would set some impossible standards for us as a society, if we have to handle every individual’s, let’s say, created crisis. What you feel as a crisis but that isn’t one. And if society has to deal with that then this is hopeless. That won’t even be possible.’

Andrea (C10): ‘I think we agree that the severity criterion is very difficult to determine from an individual perspective. Because to the individual it [their illness] will mean so much either way […] so how on earth would we place ourselves in the minds of these different people to kind of determine how they view the illness they’ve got?’

Fairness was also raised in the context of *Severity as objective fact*. Discussing the distribution of vaccines during the SARS-CoV-2 pandemic, some expressed that it was deeply unfair to consider infection in politicians and members of the royal family more severe—and therefore prioritise vaccines to them—than for other members of society. It appeared that, to ensure fairness, severity should be determined by the same objective standards across all individuals based on objective criteria pertaining to diagnoses generally.

Jon (C18) presented a different argument for ensuring fairness through objective standards, which we did not identify elsewhere in the data. He argued that considerations of how individuals handle their condition would be unfair to patients who adapt well to their illness. He seemed concerned that patients who rehabilitate well would be punished for their efforts by no longer requiring or receiving support from the healthcare system, while those who do not put in the same effort would be rewarded by receiving continued support. When discussing the idea of directing resources to those who had, across a lifespan, a greater health loss than others, and thus differentiating between individuals within patient groups, Jon (C18) stated:

Then you’d punish those who have maybe led a good and healthy life and been healthy. He’ll recover and not be as taken care of as much as the other. That can’t be right?

### Severity as situation dependent

A third theme centered on the idea that severity depends on the context surrounding the person. This emphasis on context appeared to be represented by three subthemes, based on individual circumstances, the effects of a condition on those around the person, and the impact of illness at a societal level.

#### Severity and the social effects of illness

Some expressed that severity was tied to how illness affects relational, social, and work-related circumstances. As such, severity was about how a condition affects the individual’s life in broader terms, such as the ability to parent, to work, and to enjoy hobbies or social activities. An illness or condition thereby appeared to be considered more severe if it affects one’s ability to function with it.

Caroline (C20): ‘What I’m thinking of is if you fall out of working life. Or if you fall out of hobbies you have. Or if you fall out of your social network. Then I think it’s a severe condition. Because you’re no longer, you’re not really part of normal life anymore.’

The resources surrounding an individual were also considered relevant to severity and how a condition could impact the individual. The support system surrounding a patient was one such resource, and some stated that a condition could be perceived as more severe in the absence of such a network. People’s financial situation was another example of how personal resources could modify the severity of a condition.

Sara (C16): ‘Severity maybe depends on what kind of support system you have around you […] it’s more severe to be ill if you don’t have a stable personal economy, or a lot of people around you to help. That can also affect how severe something is.’

Melissa (C20): ‘It’s less severe because she can buy herself help […] So it creates less severity when you’re resourceful.’

#### Severity and the effects of illness on others

When discussing the situational nature of severity, some related this to the effect a condition might have on those around the patient. Examples included a child affected by their parent’s illness, a family bereft of a beloved grandmother, or a social group losing a much-loved friend. A condition seemed to be considered more severe if people beyond the patient are affected.

Sandra (C19): ‘When you’re considering severity then you can’t just see the individual, you need to see everyone around […] when you’re considering the one patient you need to think about who is standing around this patient, who will suffer if you don’t prioritise it. What will happen to those around them?’

There were also suggestions this could have a cumulative effect, i.e., the more people affected, the more severe the condition. Speaking about illness generally, Mathias (C4) stated that:

I think the more people it affects, the more severe the illness is.

Parenthood appeared to be considered especially relevant, and illness in a parent could be more severe due to the effect their illness might have on their child. While talking about parenthood, Marianne (C18) expressed that the impact of losing a parent is so substantial that a life-threatening condition should be considered more severe for those with children than for those without:

I have a brother with three kids, I have no kids. I think that it’s more important that he lives than that I live, if you had to choose between us.

#### Severity and the effects of illness at a societal level

Severity was also seen as related to the effects of a condition at a societal level, with illness considered more severe if it induced negative effects on society, such as large costs associated with treatment, or a reduction in productivity. The opportunity cost of care was also pointed out as a concern at the societal level: the more resources directed towards the healthcare system, the less is available for other sectors. Emily (C19) suggested that severity also related to the implications of a condition outside the healthcare sector:

I’m relatively young, and I’m worried about the welfare state in the future, pension schemes, can we afford to treat people, can we afford to develop good enough schools, nurseries, work for everyone […] that one should focus more on prevention and trying to stop illness before it becomes too severe, before you fall out of work, so even fewer of us can contribute to the welfare state […] I think that’s also very important to consider when we’re talking about severity.

Paul (C5) pointed to the negative impact of mental health problems, beyond the effects on the patient. He argued that conditions like psychosis could be associated with increased crime rates, and that such impacts should also be considered:

The societal consequences can be enormous […] I’m thinking about the indirect consequences, that those are also part of the severity criterion. Or should be.

Fairness again came into play, but here it appeared to be associated with the relational and societal burdens of illness. David (C21) expressed concern about what was fair at a societal level:

I personally think benefit for society should also be considered. Eh, if you help someone who will lead to a greater burden for society that’s like buying yourself a problem. If you help someone who quite frankly contributes to creating goods for the community, then go for it […] My point it that the benefit for society is also important.

Rather than determining severity in a fair way for the individual as in *Severity as subjective experience*, or fairly within and across patient groups as in *Severity as objective fact*, there was an emphasis on determining severity in a manner that is fair to the wider society. As such, it appeared that determining severity fairly entails a consideration of the effects on the wider society. A fair determination of severity, it seemed, should also take into consideration what the cost of illness is at a societal level.

## Discussion

The participants provided rich, detailed, and differing descriptions of severity. We identified three interrelated conceptualisations of the term, namely *Severity as subjective experience*, *Severity as objective fact*, and *Severity as situation dependent*. The disparity between these resonates with the ambiguity surrounding severity in priority-setting literature and policy [11, 16, 17, 29], and based on the results of our analysis of lay conceptualisations of severity, the notion seems to be a complex and multifaceted one. We discuss this next, starting with what such complexity tells us about the difficulty of presenting a clear notion of what severity is. Observing that our three themes touch on central debates in the literature on priority setting generally and severity specifically, we compare our findings to positions within some of these debates. As fairness seemed central to how severity was perceived, we also discuss the different ways fairness arose in our material. Finally, we argue that there is considerable dissonance between public conceptualisations and policy operationalisations of severity.

### Severity: an inherently complex notion

While ‘severity’ is a common, everyday term, it also appears to be inherently complex. As participants discussed the multitude of issues they associated with the term in a healthcare context, they connected and contrasted different interpretations and disagreed among each other (and sometimes themselves) on exactly what severity is and how to judge whether a condition is severe or not. The disparity between the views we uncover demonstrates the difficulty of capturing what severity really is, and suggests that severity is a polyvalent concept [41], with discrepant assumptions and emphases underpinning how it is understood and applied. Furthermore, participants’ views on severity were not contained within the three analytic themes (i.e., these represent themes across participants, not individual views). Participants expressed that severity can mean many different things, and the same participant could express views aligned with more than one conceptualisation. Severity thereby appears to be a complex notion. While lay conceptualisations of severity have not been widely explored, this finding corresponds to Broqvist and colleagues’ findings of lay views on severity compared to the Swedish policy operationalisation of the term [12].

*Severity as subjective experience* and *Severity as objective fact* in many ways represent opposite ends of a continuum. The focus on the subjective and the individual, their experience of their situation, and their unique position to assess its severity is reminiscent of a phenomenological approach to illness [42–44], emphasising the role of subjectivity and how lived experience uniquely informs understandings of illness [44, 45]. Severity as an objective, criteria-based description of disease, on the other hand, bears comparison to criteria-driven approaches to healthcare [46]. These endpoints on the continuum relate to familiar positions within the wider healthcare literature and map onto a longstanding debate on whether health state evaluation should be based on individual, subjective evaluation or objective, generalisable standards [47, 48]. However, while subjective and objective approaches tend to be treated as distinct within the healthcare literature, overall our participants appeared ambivalent about the degree to which severity is subjective or objective, or whether it contains elements of both. This reinforces the notion that severity could be seen as existing somewhere along a continuum between the two.

In *Severity as situation dependent*, emphasis is on the effects of illness and where these effects are located, from considering the social effects of illness on the patient, to the effects of illness on those surrounding the patient, and finally to the effects of illness at a societal level. Some of these effects appear to be located outside the healthcare sector, beyond treatment and care. This is reminiscent of the societal perspective sometimes adopted in health economic evaluation [14, 49], where factors such as absence from (paid and unpaid) work and the burden on family members (i.e., health spillovers) are considered relevant to the decision context [50, 51]. The subthemes we identify within *Severity as situation dependent* could be said to represent three orders of effects, stretching from a first order concerning the patient and the social effects of illness on them, to a second order of effects of illness on those immediately surrounding the patient, and finally to a third order of the broader effects of illness at a societal level. It is notable that priority-setting frameworks tend to adopt a healthcare perspective when considering cost-effectiveness evidence, and severity is often operationalised as disregarding the effects of illness beyond the patient and their medical needs [4, 7]. The orders of effects within *Severity as situation dependent*, however, demonstrate a concern among the citizenry for the relevance of indirect burdens and costs of illness to severity.

### Determining severity fairly

In all three analytic themes, the issue of fairness arose as relevant to how, and on what level, severity is determined. In *Severity as subjective experience*, concern is with fairness for the individual patient. In *Severity as objective fact*, emphasis is on ensuring a fair, independent determination of severity across all those using health services. In *Severity as situation dependent*, there is a concern for determining severity fairly at a societal level.

Concern for ensuring a fair determination of severity both for the individual and for society also compares to the literature on health economic evaluation, specifically within the literature on QALYs themselves and who ought to evaluate health states [10, 14, 52, 53]. QALY estimates (more specifically, the valuation of health states, which are used to estimate QALYs) commonly rely on preferences elicited from members of the public [54] so that, when severity is operationalised via QALYs, a condition’s severity is determined by the preferences of members of the public who do not suffer from that condition. A central argument for employing public preferences in QALY estimations is that, because public funds pay for healthcare, it is fair that the public should determine the relative value of different health states [53]. This argument is comparable to the way fairness arose in the subtheme of *Severity as situation dependent* concerning societal effects, emphasising the importance of determining severity fairly on a broader, societal level. In *Severity as subjective experience*, on the other hand, emphasis was on ensuring fairness by determining severity according to the individual’s subjective experience. This aligns with a common critique of public preference-based QALY estimation and ties back to the notion of lived experience, i.e., that patients know their condition best, and are therefore best situated to evaluate it [53].

The emphasis within *Severity as objective fact* on applying criteria to determine severity fairly within and across patient groups is somewhat aligned with a central motivation behind the QALY, namely to create a standardised approach to classifying health states across patient groups [55]. Here, we also identified a notion of fairness centred on determining severity objectively to avoid punishing those who adapt well to their illness. This view, which was expressed by one participant, stands in stark contrast to the egalitarian principle of concern for the worse-off central in health economic literature, asserting that patients who fail to adapt to illness should not be punished for it [56–59]. This alternate notion of fairness, which could be interpreted to voice concern for the better-off, thereby compares to a libertarian position [60] and represents an antithetical approach to severity and fairness to established norms and policies in public healthcare systems.

### Conceptual and operational mismatch

Our results touch on many central debates and contested issues in the priority-setting literature generally and on severity and QALYs specifically. This is an interesting finding in itself, demonstrating that these debates are not exclusive to the domains of policy and academia, but relate to issues members of the public intuitively care about and point to. Reflecting on the issues brought up in the conversations, there are elements across the analytical themes that might be supported by QALY-based operationalisations of severity, such as the relevance of the risk of death, illness prognosis, and quality of life. However, our results touch on a plethora of additional concerns and appear to contain more than QALY shortfall outcomes. For example, we demonstrate views linked to the relevance of lived experience, and determining severity in a way that is fair to the individual. We observe concern for the non-health related effects of illness, as well as for effects on family members and friends. And we identify approaches to severity from a societal perspective, including concern for production loss. These additional concerns are not accounted for in the Norwegian, Dutch, or UK policy operationalisations of severity, based on absolute and/or proportional QALY shortfall [6, 7, 9]. Many of these concerns are also unaccounted for across the somewhat broader, qualitative severity levels within the Swedish model [5]. This supports our claim that severity is a polyvalent concept which is not neatly defined, applied, or contained. Participants’ reluctance to describe any condition as definitively non-severe further complicates the use of severity in policies: not only is it difficult to say exactly what severity is; it also appears difficult to say what it is not.

The healthcare systems we refer to above, which employ severity in healthcare policies, have a long tradition of working systematically with priority-setting; priority-setting is complex, and outcomes have a considerable impact on people’s healthcare provision, and lives. The time-consuming processes of establishing priority-setting criteria reflect this complexity. However, the operationalisations of severity those processes have culminated in represent narrower interpretations of severity than those members of the public appear to hold. Severity is a complex term, and while the varied views on the meaning of illness severity we identify reflects this complexity, we argue that current operationalisations of severity do not appear to account for the multifaceted nature of the term. Our findings thus suggest there is poor alignment between operationalisations of severity in policy and conceptualisations held by members of the public. Such a mismatch can make it difficult for members of the public to understand and support priority-setting decisions involving the term severity. This could lead to complaints from the public on priority-setting outcomes, and policymakers therefore ought to ensure that operationalisation of terms used in everyday parlance corresponds to everyday meanings of them when such terms are applied in policies. Our findings suggest members of the public might not consider current policy operationalisations to sufficiently capture the meaning(s) of illness severity in a healthcare context.

### Strengths and limitations

A strength of this study is the breadth of the sample with regards to age, sociodemographic background, and geographical location. Despite considerable efforts, we recruited few participants with minority backgrounds, and there is an overrepresentation of women and individuals with higher education in the sample. The majority also reported to be in good health, on average somewhat better than the Norwegian population more widely [61]. It is possible that a broader and larger sample would have yielded additional perspectives. Due to the SARS-CoV-2 pandemic restrictions, the conversations varied in format (online/in-person) and size. This may have influenced the way in which the conversations progressed. The group setting may also have influenced the degree to which participants felt free to express their personal views. We strived to both identify and avoid interpretive biases [62]. As preconceptions could influence interpretations, we sought to avoid biases in coding and analysis by having three authors code the same three transcripts and compare approaches. The analytical process was also a collaborative and reflexive process between all authors. The research was conducted in Norway, and results may not be applicable in other contexts, even if severity is used in other jurisdictions as a priority-setting criterion.

## Conclusion

Having explored the knowledge gap on what severity means to members of the public, the three interrelated conceptualisations we identify suggest that severity is a polyvalent concept. Comparing our findings to the literature on priority setting and severity, it is evident that, while there is some overlap with QALY-based operationalisations, severity appears to involve many additional concerns for the citizenry. Our findings provide policymakers with a richer understanding of what severity means to members of the public and demonstrate that there appears to be a dissonance between public conceptualisations and policy operationalisations of severity.

### Electronic supplementary material

Below is the link to the electronic supplementary material.

Supplementary Material 1

Supplementary Material 2

Supplementary Material 3

## Data Availability

The dataset generated and analysed during the current study are not publicly available due to privacy concerns for the participants, but are available from the corresponding author on reasonable request.

## Abbreviations

QALY Quality: adjusted life year
SEVPRI: SEVerity and PRIority setting in healthcare
UK: United Kingdom
US: United States
