# Supplementary material for: “It’s hard to say anything definitive about what severity really is”: lay conceptualisations of severity in a healthcare context
Source: BMC Health Serv Res. 2024 Apr 19;24:490. doi: 10.1186/s12913-024-10892-6 (PMC11031975; doi:10.1186/s12913-024-10892-6)
Supplement: Supplementary file 1 — Supplementary Material 1 [file 12913_2024_10892_MOESM1_ESM.pdf]

Additional File 1: Conversation Guide (introductory text for conversations and topic guide). The version presented here is an author's own English translation of the original Norwegian version, translated for the purpose of publication.

Conversations Guide

Subjective views on severity

The conversations are conducted in the format of open conversation, with the participants leading the conversations. Facilitators ask some follow-up questions, asking participants to explain their viewpoints, and also noting down new topics that arose. The course of the conversations is illustrated in Figure 1 below.

Figure 1 An illustration of the course of the conversations. The text in the illustrations is a non-validated authors-own translation for dissemination purposes only.

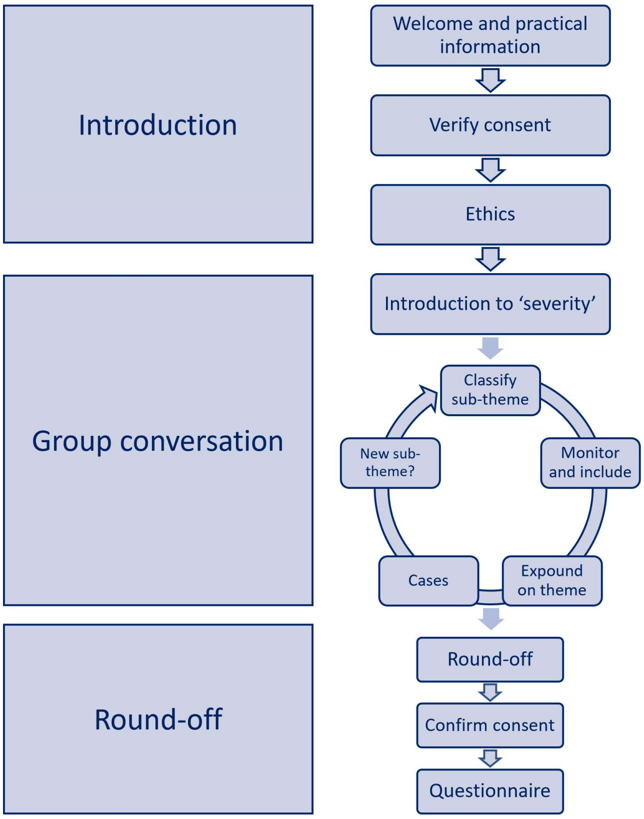

Welcome and practical information

Welcome

Health and safety: Where are the toilets? Escape routes. Option to leave the conversation.

Practical: 3 hours planned. The plan for food/drinks. Distribution of papers and pens. Remuneration.

Ethics/Consent

- Distribution and review of consent form.
- Ask participants to have mobile on silent/flight mode, no audio recordings or pictures.
- Information regarding confidentiality.

Read

Thank you for being willing to contribute to this project. By participating in this focus group you will contribute to research on healthcare priority setting in Norway. During the conversation, some topics may arise that some feel uncomfortable discussing, or that lead to feelings of anxiousness. We understand that this can be difficult and want to emphasise that you can leave the group if you wish to. *The conversation will be recorded and then transcribed, and anonymised*. These data will be securely stored and the anonymised material will only be available for the project collaborators, *as described in the consent form*. *[CHECK CONSENT FROM EVERY PARTICIPANT]*. You have a duty of confidentiality concerning what you learn about other participants, and it is prohibited to record sound or images of each other during the conversation. Do you have any questions so far?

Introduction to the group conversation on SEVERITY

- Provide introduction to the topic of severity and context for why this is a relevant issue to explore.

Read (introducing the theme)

Norwegian priority-setting guidelines state that the cost of treatment and the benefit, i.e. the health benefit, for the patient are important factors that should be taken into account. In addition, there is a *severity criterion*.

In simplified terms, the severity criterion states that we should prioritise saying ‘yes’ to treatments that are actually “too expensive” if the condition that is being treated is “severe enough”. *[pause]*

This also means that one should say ‘no’ to treatment for less severe conditions if it is too costly. *[pause]*

We don’t know exactly what the population think *severity* means. We also don’t know what role the population think severity should have in healthcare priority setting.

Guidelines for the conversation

Read

Before you begin to discuss severity, we would like to emphasise that there are many different viewpoints on this topic. We do not think there are any right or wrong views on this. Health personnel, economists, and philosophers also discuss what severity is, and what role severity should play in priority setting.

The purpose of this focus group is to explore the different *subjective* viewpoints in the population in Norway. All thoughts and input, thought through or spontaneous, clear ideas or vague impressions, based on personal or professional experience, are equally useful, important, and welcome today.

We want you to talk to each other rather than to us, and we will first and foremost be moderators. We will nonetheless participate in the conversation, and may provide input and interpretations, or follow-up questions if we wish to hear more about something.

So: what do you think severity is? Begin with the first associations that come to mind, and then we can talk more about priority setting later on.

Topic guide (updated dynamically)

There are some topics we expect to arise in the conversations, based on a literature review conducted ahead of this study (1), which identified common and relevant concerns. Not all the topics need to be discussed in every conversation, but form a starting point for discussions. These topics include:

- Age
- Physical pain
- Mental discomfort
- Quality of life
- Identify
- Dignity
- Social context and role
- Level of functioning
- Prognosis at time of diagnosis
- Chronicity
- Progressive disease (ALS, Alzheimer, Parkinson)
- Death
- Hope and hopelessness
- Narrative vs. episodic
- Degree of urgency
- Risk of future severity (prognosis)
- Preferences/individual considerations
- Condition vs. illness vs. situation
- Desert
- Lifetime perspective vs. future perspective
- Relative/absolute (relative to other patients, relative to different dimensions)
- Second order effects
