# Supplementary material for: “It’s hard to say anything definitive about what severity really is”: lay conceptualisations of severity in a healthcare context
Source: BMC Health Serv Res. 2024 Apr 19;24:490. doi: 10.1186/s12913-024-10892-6 (PMC11031975; doi:10.1186/s12913-024-10892-6)
Supplement: Supplementary file 2 — Supplementary Material 2 [file 12913_2024_10892_MOESM2_ESM.pdf]

Additional File 2: A copy of the questionnaire that was filled out by participants following the conclusion of conversations. The version presented here is an author's own English translation of the original Norwegian version, translated for the purpose of publication.

Conversation on Severity

Thank you for filling out this form. All the information we collect is for research purposes. Information will be unidentified and deleted after the project ends. The purpose of this form it to be able to describe the participants as a group, and to better be able to interpret the meaning of the conversation you have participated in.

Questionnaire number (RID):	
Date:	
Project collaborator:	

I CONSENT TO RESPOND TO THIS QUESTIONNAIRE AND TO MY RESPONSES BEING STORED AND USED AS DESCRIBED.
(THIS CONSENT IS NOT VALID WITHOUT A SIGNED CONSENT FORM FOR THE GROUP CONVERSATION ABOUT SEVERITY.)

Place and date

Participant signature

Participant name in capital letters

QUESTIONNAIRE

PART I – ABOUT YOU

Questions 1–12 allow us to describe the participants in these group conversations. In addition, this information helps us to recruit future participants. In this phase of the study, we wish to meet as many different people as possible, and in this way we identify the breadth in the participants' background. These responses also help us to interpret the meanings within the conversations. Your responses will be saved unidentified and separate from directly identifiable data like name and contact information.

S1 **Sex**

Female	Male	Other/Prefer not to say
☐	☐	☐

S2 **Age**

- ☐ 18-30
- ☐ 31-50
- ☐ 51-66
- ☐ 67+
- ☐ Prefer not to say

S3 **What is your highest education level**
(One answer only.)

- ☐ Elementary school
- ☐ Upper secondary school
- ☐ Fagbrev
- ☐ Bachelor degree
- ☐ Master degree
- ☐ Profession study (MD, dentist etc.)
- ☐ PhD
- ☐ Other (please describe) ____________
- ☐ Prefer not to say

S4 **If you live with someone, state the number within each age category.**

Children (0 – 16 years old)	
Adults (17 – 66 years old)	
Elderly (> 67 years old)	

☐ I am married/have a cohabitant

☐ Prefer not to say

S5 **Do you consider yourself religious or spiritual?**

(Mark the alternative that best suits you.)

Religious/spiritual *Active in a community*	Religious/spiritual *Not active in a community*	No	Prefer not to say
☐	☐	☐	☐

S6 **Have you or anyone you know had a severe illness?**

(Several marks are possible.)

☐ Yes, passing

☐ Yes, chronic

☐ Yes, with deadly outcome

☐ Don’t know

☐ No

☐ Prefer not to say

If you responded ‘Yes’ to **Question 6**, please elaborate in **Question 6b**

S6b **Which relation did you have to that person?**

(Several marks are possible.)

☐ Partner

☐ Parent

☐ Child

☐ Sibling

☐ Friend

☐ Other family member (please describe)

☐ Other (please describe)

☐ Prefer not to say

S7 **How do you consider your own health?**
(One answer only.)

Very good	Good	Okay	Poor	Very poor	Don’t know	Prefer not to say
☐	☐	☐	☐	☐	☐	☐

S8 **Generally, what is your impression of the quality of the Norwegian public healthcare system?**
(One answer only.)

Very good	Good	Okay	Poor	Very poor	Don’t know	Prefer not to say
☐	☐	☐	☐	☐	☐	☐

PART II – ABOUT THE CONVERSATION

S9 **What would you say has had the greatest influence on your view of what *severity* means in relation to illness?**
(Use the numbers 1, 2 and 3 to rank the most important ones.)

- ☐ My upbringing
- ☐ Media
- ☐ Religious views/Outlook
- ☐ Family
- ☐ Education
- ☐ Political views
- ☐ Life experience
- ☐ Prefer not to say
- ☐ Work/profession
- ☐ Single event in life (please describe)
- ☐ Organisations (please describe)
- ☐ Other (please describe)
- ☐ Don’t know/Difficult to say

We will not identify you when we report on our findings in this study, but we wish to convey something about the background of participants to better illustrate the subjective viewpoints that have been expressed in these meetings.

S10 **If you have any suggestions as to how we can describe you, you can write it in the section below. You can tie the description to what you think might have influenced what you have expressed in the group conversation. What would you say is your field of expertise? Is there something else that it is important to know?**
(For example: 'disabled nurse', 'lawyer and mother', 'man in his thirties with responsibility for sick father'.)

S11 **We would like to speak to as many people as possible. The goal is to identify as many viewpoints on severity as possible. Is there 'someone' you think might have valuable perspectives on this?**
(For example: 'minimum pensioners', 'immigrants', 'cancer patients')

S12 **How do you feel the group conversation went?**
(Did the facilitator participate too much or too little? Were examples that the facilitators mentioned useful? Did you feel that you got the chance to speak?)

Thank you for your help!
