# Supplementary material for: “It’s hard to say anything definitive about what severity really is”: lay conceptualisations of severity in a healthcare context
Source: BMC Health Serv Res. 2024 Apr 19;24:490. doi: 10.1186/s12913-024-10892-6 (PMC11031975; doi:10.1186/s12913-024-10892-6)
Supplement: Supplementary file 3 — Supplementary Material 3 [file 12913_2024_10892_MOESM3_ESM.pdf]

Additional File 3: Consent form. The version presented here is an author's own English translation of the original Norwegian version, translated for the purpose of publication.

WOULD YOU LIKE TO PARTICIPATE IN THE RESEARCH PROJECT SUBJECTIVE VIEWS ON SEVERITY?

AIM OF THE PROJECT AND WHY YOU'RE BEING ASKED

This is a question to you about participating in a research project where we wish to know more about which different subjective views on the term "severity" exist in the Norwegian population.

The term severity is central in Norwegian healthcare priority setting. How severity is interpreted and used in decisions regarding priority setting in the healthcare system has considerable consequences for healthcare budgets, public spending, and the individual patient. The research project is financed by the Research Council of Norway (project number 303724 - SEVPRI) and is called *subjective views on severity*.

We therefore wish to speak to different people in different regions of Norway. Recruitment is conducted through posters, project collaborators' networks and social media.

WHAT DOES THE PROJECT INVOLVE FOR YOU?

First and foremost, participation in the project involves participation in a guided group conversation about "severity". The conversation will last for a maximum of 3 hours, and there will be a break for a light meal. You can at any point, without providing a reason, leave the group meeting. We compensate participation before the meeting commences with a sum of 500 Norwegian kroners for time, loss of income, and travel expenses.

During the meeting, we will audio record the group discussions. Audio recordings will be saved as encrypted files or stored in a safe in a restricted-access room at Akershus University Hospital Trust (Ahus). The audio recordings will not at any point be saved or listened to from equipment with internet access. The audio recordings will be transcribed, and the transcriptions will be saved on a digital medium and as paper files in a safe. In the transcriptions, it will not be possible to connect what you have said to your identity.

You may hear about personal and sensitive experiences from other conversation participants. By participating, we expect you to consent to confidentiality concerning what other participants share.

After the group conversation we will ask you to fill out a questionnaire with background information about yourself. The background information we would like you to share includes gender, age, household size, and some questions about experiences with illness in your close family, religion, and personal health. It is voluntary to fill out to the questionnaire.

It may become relevant for us to contact some participants by telephone or email for follow-up questions. We ask for your separate consent to being contacted. If you give us your consent to contact you for follow-up questions at a later point, this will typically take 10-15 minutes. You can reject such contact later even if you give us permission to contact you. All follow-up conversations are planned to be completed by the end of December 2021.

POTENTIAL PROS AND CONS

+ Many participants in qualitative studies find it meaningful to contribute to new knowledge with their experiences. You will also gain new insights into how other people think about the topic. Participation in this project also gives you the opportunity to affect the academic discourse on priority setting in the healthcare services in Norway. This gives you a voice in Norwegian priority-setting work.

- There is always a possibility that information can get lost. We believe the information handling procedures in SEVPRI are good, and that the risk of information you provide us with getting lost are minimal.

- Participating in a conversation about severity can include discussion of topics that are sensitive. Some may therefore experience this kind of meeting as stressful or uncomfortable.

- Though papers based on qualitative interviews seek to anonymise participants, it is possible that you may recognise your own statements in the academic treatment and description of our data. As researchers, we seek to maintain a neutral and descriptive role when we describe statements and opinions that have emerged. Research ethics guidelines[1,2] hold that we who have spoken to you must do our best to treat the content of the conversations with respect and without judgement. Still, it may happen that participants in qualitative studies are left with a sense of being misunderstood, misinterpreted, or incorrectly sited. This may lead to a sense of having been offended or violated. The researchers who will be working with the content of the conversations will work without judgment and have a high degree of awareness on this issue.

VOLUNTARY PARTICIPATION AND POSSIBILITIES FOR REVOKING CONSENT

Participation in the project is voluntary. If you wish to participate you can sign the consent form on the next page of this document. You can at any point and without providing a reason pull out from the meeting, from filling out the questionnaire, and from further participation. This will not have any negative consequences for you.

You can at any point and without providing a reason revoke your consent. It will not have any negative consequences for you if you later decide to pull out.

If you later wish to pull out or have any questions about the project, you can contact the project leader (see contact information on the next page). If you revoke your consent, your personal information will not be used for further analysis. Requests for destruction or deletion of data is not valid if the material or information is anonymised. Such requests can also be denied if the data forms part of completed analyses, or if the material has been processed and is represented only in summary or aggregated form. Specifically, this means that if SEVPRI has reported that "the conversation participants consisted of 40% women and 60% men", we will not adjust these number if you decide to pull out.

WHAT HAPPENS TO THE INFORMATION ABOUT YOU?

The information that is registered about you will only be used as described under the aims of the project, and will be analysed in the project period 2020—2024. Project closure is planned for 31.12.2024. Potential extension of the project period beyond this date will only occur according to approval of the Data Protection Officer at Ahus, and such an extension will only pertain to the duration of the project period.

You have a right to insight into what information is registered about you and the right to correct potential mistakes in the registered information. You also have the right to insight into the security procedures concerning

[1] https://www.forskningsetikk.no/retningslinjer/med-helse/vurdering-av-kvalitative-forskningsprosjekt-innen-medisin-og-helsefag/
[2] https://www.forskningsetikk.no/retningslinjer/hum-sam/forskningsetiske-retningslinjer-for-samfunnsvitenskap-humaniora-juss-og-teologi/

the treatment of that information. You can complain about the treatment of your information to the Data Protection Authority and the Data Protection Officer at Ahus.

All information will be treated without name, personal ID number, or other directly identifying information (coded information). A code connects you to you information via a name list. Only the project leader (Mathias Barra) and the PhD candidate on the project (Mille Sofie Stenmarck) have access to this list.

The information that is collected will for control purposes be stored for five years after project closure, before they are deleted.

FOLLLOW-UP STUDIES

It may become relevant for project collaborators of SEVPRI to contact you for further discussions of the term severity. By providing your consenting here, and by also providing your email and/or phone number, you give SEVPRI permission to contact you via email and/or telephone. You are not required to participate in a potential follow-up study, but give us the permission to contact you with the request of such a follow-up. Your contact information will be stored with, and with the same handling procedures as, the name list.

FINANCE

You will receive 500 Norwegian kroners as compensation for travel expenses and time spent on participating in the group conversations. The sum will be paid out as a universal gift card when you sign this consent form.

SEVPRI is partly financed by the Research Council of Norway (project number 303724). None of the project collaborators have any financial interests in this study.

APPROVALS

The Regional Committee for Medical and Health Research Ethics (REK Southeast B) has completed a research ethics assessment (ref. no. 186284) and found that SEVPRI is not regulated by the Health Research Act. Therefore, the Data Protection Officer at Ahus has considered the project and advised that the project can commence.

Akershus University Hospital Trust and project leader Mathias Barra are responsible for research ethics and privacy concern in the project.

CONTACT INFORMATION

If you have questions relating to the project or wish to withdraw from participation, you can contact SEVPRI at sevpri@ahus.no or Mathias.Barra@ahus.no.

If you have questions regarding privacy concerns in the project, you can contact the Data Protection Officer at Ahus:

Email: personvern@ahus.no; www: https://www.ahus.no/avdelinger/personvernombud

The email address of the Data Protection Authority is postkasse@datatilsynet.no; phone 22 39 69 00;

www: https://www.datatilsynet.no/om-datatilsynet/kontakt-oss/

I CONSENT TO (1) PARTICIPATING IN THE PROJECT, (2) TO MY PERSONAL INFORMATION BEING STORED AND USED AS DESCRIBED ABOVE, AND (3) TO OBSERVING MY DUTY OF CONFIDENTIALITY. *BOTH (1), (2), AND (3) ARE NECESSARY TO PARTICIPATE.*

Place and date

Participant's signature

Participant's name in capital letters

I HAVE RECEIVED A GIFT CARD OF 500 NOK

Place and date

Participant's signature

I CONSENT TO BEING CONTACTED BY SEVPRI AGAIN BY 1ST AUGUST 2021

☐ Email

☐ Phone

(Mark the relevant boxes)

Participant's signature

I CONFIRM THAT I HAVE PROVIDED INFORMATION ABOUT THE PROJECT

Place and date

Signature

Role in project
